# Diagnostic value of anti-Kaiso autoantibody in axial spondyloarthritis

**DOI:** 10.3389/fimmu.2023.1156350

**Published:** 2023-03-30

**Authors:** Xinzhe Feng, Wenwen Tong, Jia Li, Yihong Xu, Shanbang Zhu, Weidong Xu

**Affiliations:** Department of Joint Bone Disease Surgery, Changhai Hospital, Navy Medical University, Shanghai, China

**Keywords:** axial spondyloarthritis, biomarker, autoantibody, Kaiso, diagnosis

## Abstract

**Objective:**

Axial spondyloarthritis (axSpA) is a chronic rheumatic disease predominantly characterized by inflammation and progressive structural damage. Patients are often diagnosed very late, which delays the optimal treatment period. Early diagnosis of axSpA, especially non-radiographic axSpA (nr-axSpA), remains a major challenge. This study aimed to investigate the diagnostic value of anti-Kaiso autoantibodies in axSpA and their correlation with clinical disease indicators.

**Methods:**

Two pooled serum samples (seven patients with nr-axSpA and seven healthy controls) were profiled using HuProt arrays to investigate the diagnostic value of autoantibodies in nr-axSpA. Levels of anti-Kaiso autoantibodies in patients with axSpA and controls were determined using the Meso Scale Discovery assay system. Receiver operating characteristic curve analysis was performed to evaluate the diagnostic performance of anti-Kaiso autoantibodies in axSpA. Pearson’s correlation was used to assess the correlation between anti-Kaiso autoantibodies and clinical parameters.

**Results:**

Seven candidate autoantibodies were present in the serum of patients with nr-axSpA. The levels of anti-Kaiso autoantibodies were significantly higher in the nr-axSpA group than in the other groups. It can differentiate nr-axSpA from ankylosing spondylitis (AS), healthy controls, and rheumatoid arthritis. The level of early-stage AS among patients with nr-axSpA decreased when they progressed to the late stage. Of all patients with axSpA, serum anti-Kaiso autoantibody levels were positively correlated with the C-reactive protein level and the Bath Ankylosing Spondylitis Disease Activity Index score and negatively correlated with disease duration.

**Conclusion:**

Anti-Kaiso autoantibody may be a valuable diagnostic biomarker for early-stage AS in the nr-axSpA period and may be a potential therapeutic target.

## 1 Introduction

Axial spondyloarthritis (axSpA) is widely considered a chronic, debilitating, inflammatory disease characterized by clinical signs, such as progressive spinal ankylosis, sacroiliitis, uveitis, and enthesitis (1). Owing to the lack of biomarkers and typical early symptoms, it can take 8–10 years after the onset of the first clinical symptoms to reach a definitive diagnosis (2). Inflammatory back pain and sacroiliitis are the characteristic manifestations of axSpA. As the disease progresses, structural damage becomes more severe, and the spine and peripheral joints become involved. Progressive spinal ankylosis eventually results in significant impairments in physical function, working ability, and quality of life (1). Early diagnosis of axSpA is of the utmost importance for early therapeutic intervention, which is essential for improving the long-term outcome of the disease (3).

AxSpA is commonly viewed as a seronegative disease due to the absence of rheumatoid factors (4). B cells and autoantibodies play important roles in the pathogenesis of axSpA (5). Several studies have revealed the prognostic value of serum autoantibodies, and various antigens have been identified as the sources of these autoantibodies, including microbes, inflammatory targets, and the skeletal/connective tissues (5–7). These antigens play a vital role during axSpA. Microbes and inflammatory targets may be related to the inflammatory process, and autoantibodies against the skeletal/connective tissue can lead to local inflammation, bone destruction, and even new bone formation in axSpA (7).

Structural damage in axSpA incorporates aspects of bone destruction and new bone formation, which are often the result of early inflammation. The current evidence implies that when the structural damage appears to be of a mild extent that often cannot be captured using radiology, clinical intervention could achieve the optimal effect. However, the pathogenesis involved in the structural damage of axSpA is complex and not fully understood (1, 8). Bone morphogenetic proteins (BMPs) and wingless proteins (Wnt) are key pathways driving structural damage in ankylosing spondylitis (AS) (8). Recently, the possible roles of autoantibodies and their corresponding antigens in new bone formation have been explored (9). Thus, the diagnostic value of autoantibodies in non-radiographic axSpA (nr-axSpA) and their possible roles in early inflammation and structural damage require further investigation.

In this study, we extensively profiled autoantibodies in the serum of patients with nr-axSpA and healthy controls using human proteome microarrays. We then validated that anti-Kaiso autoantibodies were higher in patients with nr-axSpA than in other controls (AS, rheumatoid arthritis [RA], and healthy controls). Furthermore, we found no significant difference in the expression of the Kaiso protein in the synovial and ligament tissues of patients with AS and RA. In addition, we investigated the prognostic value of anti-Kaiso autoantibodies in patients with axSpA and nr-axSpA. The relationship between anti-Kaiso autoantibodies and clinical characteristics was also evaluated.

## 2 Materials and methods

### 2.1 Biological samples

Serum samples were obtained from patients recruited from the outpatient clinic of Changhai Hospital (Shanghai, China). Synovial and ligament tissues were obtained from patients with AS and RA who underwent surgical treatment (spinal osteotomies or hip replacements) at the late stage of the disease. Puncture biopsy samples were obtained from the sacroiliac joints of the nr-axSpA patients. The study was approved by the Medical Ethics Committee of Changhai Hospital, and informed consent was obtained from all patients. The patients were classified according to the Assessment of SpondyloArthritis International Society criteria (10). Patients with AxSpA were divided into two subgroups (AS and nr-axSpA), and patients with AS were classified according to the modified New York criteria (11). All patients with nr-axSpA were followed up. Patients with RA fulfilled the American College of Rheumatology’s revised criteria (12). Healthy controls and patients with RA should not have any disease involving the axial joint. Disease activity and functional capacity were determined based on the Bath Ankylosing Spondylitis Disease Activity Index (BASDAI) and the Bath Ankylosing Spondylitis Functional Index (BASFI), respectively. Syndesmophytes in AS with axial involvement were assessed according to the modified Stoke Ankylosing Spondylitis Spinal Score (m-SASSS) by two experienced radiologists who were blinded to the patients’ clinical data (13). The final score is the average score. The erythrocyte sedimentation rate (ESR) (mm/h), C-reactive protein (CRP) (mg/L), and serum alkaline phosphatase (ALP) (U/L) levels were measured at the time of the assessment.

### 2.2 Serum profiling with human proteome arrays

The HuProt microarrays used in this study were provided by CDI Laboratories Inc. (Mayaguez, USA). The array contains duplicate spots of approximately 20,000 individually purified human proteins with an N-terminal glutathione S-transferase (GST) tag and is widely used to screen autoantibodies in various diseases (14, 15). Autoantibodies in nr-axSpA were profiled using HuProt arrays of two pooled serum samples (seven nr-axSpA *vs*. seven healthy controls) according to the manufacturer’s protocol.

### 2.3 Measurement of anti-Kaiso autoantibody using the meso scale discovery assay system

Levels of anti-Kaiso autoantibody in all included patients with axSpA (nr-axSpA and AS) and controls (RA and healthy controls) were determined using the MSD assay system, which is based on the detection of the light emitted by an electrochemiluminescent Sulfo-tag label following electrochemical stimulation (16, 17). The concentration of coated recombinant protein, dilution of serum and standard antibody, and amount of Sulfo-tag-labeled IgG used in the experiment were all optimized in a series of preliminary experiments. A mouse monoclonal anti-Kaiso antibody (Abcam, Cambridge, UK) was used as the internal standard positive control in the assay, and a standard curve was generated using serial dilutions (2,500, 625, 156.25, 39.06, 9.77, 2.44, 0.61, and 0 ng/ml) of the Kaiso antibody. The 96-well plates (MSD, Rockville, Maryland, USA) were then coated with 40 µl (5 µg/ml) of recombinant human Kaiso protein (Abcam) and incubated overnight at 4°C. The plates were washed three times with PBST (PBS + 0.05% Tween 20) and blocked with Blocker A (MSD) at room temperature (RT) for 2 h. After washing with PBST, 50 µl plasma (diluted 1:250 in PBS) was added to the coated plates and incubated for 2 h at RT. After washing the plates three times with PBST, 50 µl (1 µg/ml) of Sulfo-tag goat anti-human IgG secondary antibody (MSD) was added to each well and incubated for 2 h at RT on a shaker. After three more washes, 150 µl MSD read buffer (MSD) was added to each well, and the plates were read using an MSD Sector Imager S600. The serum antibody concentrations were determined by referencing the enhanced chemiluminescence (ECL) responses against the standard curve.

### 2.4 Immunohistochemistry

Synovial and ligament tissues were fixed in 4% paraformaldehyde, embedded in paraffin, and sliced into sections. The sections were then de-waxed, hydrated, and washed. After antigen retrieval using microwaves, endogenous peroxidase activity was blocked in a 3% H2O2 solution. The sections were subsequently incubated overnight with anti-Kaiso antibodies or control IgG (Proteintech, Rosemont, USA). They were then washed and incubated with horseradish peroxidase-conjugated secondary antibodies, followed by treatment with diaminobenzidine (DAB) to visualize the signal. Sections were examined and photographed using an Olympus BX51 microscope (Olympus Corporation, Tokyo, Japan).

### 2.5 Statistical analysis

Statistical analyses were performed using SPSS Statistics software version 19.0. All data were analyzed using a normal distribution test. A comparison of measurement data with a normal distribution was performed using the Student’s t test. A comparison of measurement data with non-normal data was performed using the Mann–Whitney U test. A receiver operating characteristic (ROC) curve analysis was performed to evaluate the diagnostic performance of anti-Kaiso antibodies in AS. The optimal cut-off values in the ROC curves were defined as the points at which sensitivity and specificity were maximized. The positive predictive value (PPV) and negative predictive value (NPV) were calculated. Pearson’s correlation was used to assess the correlation between anti-Kaiso autoantibodies and other clinical parameters. Logistic regression was used to analyze the anti-Kaiso antibody level and its associated clinical indicators in patients with axSpA. Differences were considered statistically significant at *P <0.05 and **P <0.01.

## 3 Results

### 3.1 Detection of autoantibodies in nr-axSpA serum with human proteome microarrays

**Figure 1:**
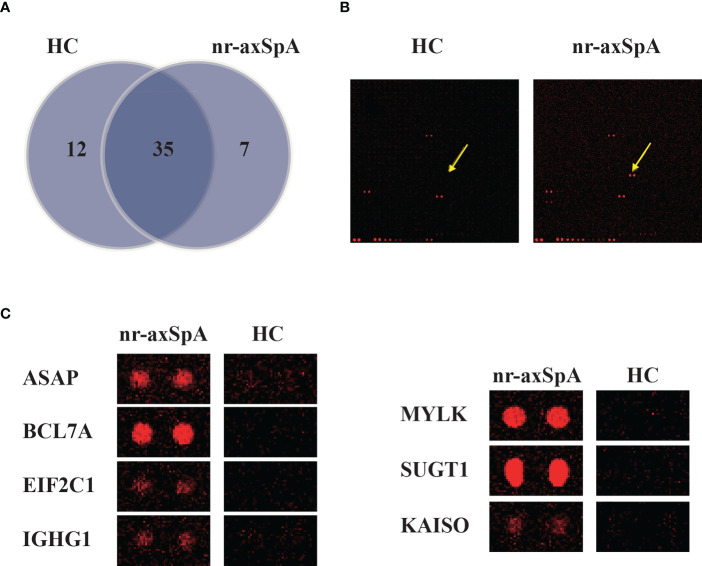
Identification of autoantibodies in the serum of patients with nr-axSpA using human proteome (HuProt) microarrays. **(A)** The Venn diagram shows the number of autoantibodies identified in the serum of patients with nr-axSpA and healthy controls. **(B)** Representative images of the autoantibody detected in the HuProt microarrays. The arrows indicate the duplicate spots of the antigen protein. **(C)** High magnification images of the seven specific autoantibodies, including ASAP, BCL7A, EIF2C1, IGHG1, MYLK, SUGT1, and KAISO, identified in patients with nr-axSpA.

We used the HuProt microarray to identify autoantibodies in two pools of samples collected from patients with nr-axSpA (n = 7) and healthy controls (n = 7). Data analysis revealed that 47 autoantibodies were detected in the serum of healthy controls and 42 autoantibodies were found in the serum of patients with nr-axSpA. Further analysis showed that seven candidate autoantibodies were specifically present in the serum of patients with nr-axSpA but not in healthy controls (**Figure 1A**). Representative images of the autoantibodies detected on HuProt microarrays are shown in **Figure 1B**. High-magnification images of the seven specific autoantibodies (ASAP, BCL7A, EIF2C1, IGHG1, MYLK, SUGT1, and KAISO) are presented in **Figure 1C**.

### 3.2 Validation of novel autoantibodies using the MSD assay system

**Figure 2:**
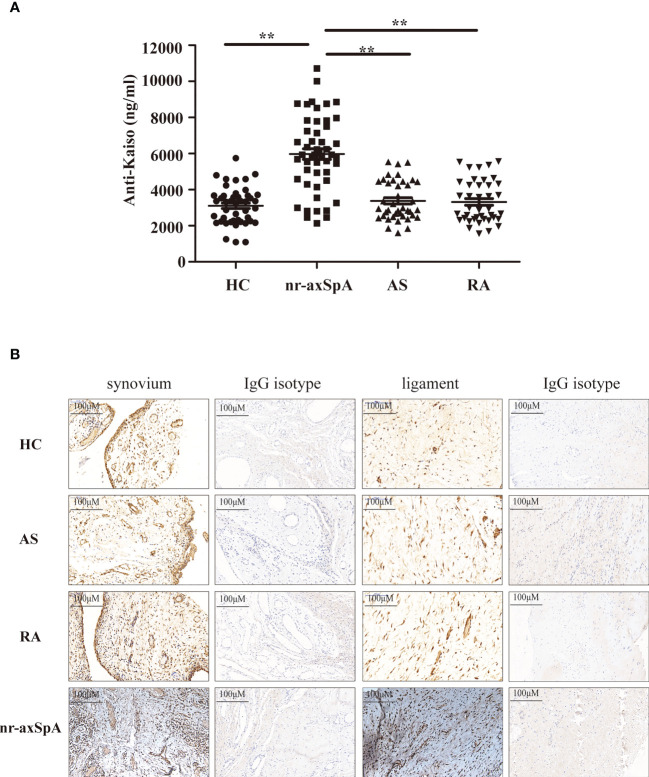
Validation levels of anti-Kaiso autoantibodies in the serum using the Meso Scale Discovery (MSD) assay system and the expression of Kaiso in the tissues detected using immunohistochemistry (IHC). **(A)** MSD analysis of anti-Kaiso autoantibodies in the serum of patients with nr-axSpA (n = 50), AS (n = 40), and RA (n = 40) and healthy controls (HC, n = 45). **(B)** Synovial and ligament tissues of HC, AS, RA, and nr-axSpA were stained with the anti-Kaiso antibody. Data are presented as mean ± SEM. **P <0.01 compared to patients with nr-axSpA. All IHC images are shown at ×100 magnification and are representative of images from three independent experiments.

As inflammation and progressive new bone formation are hallmarks of axSpA, especially in AS, we focused on Kaiso-specific autoantibodies, which have been shown to affect inflammation and Wnt signaling (18, 19). We measured the amount of anti-Kaiso antibodies in serum samples using MSD in a large cohort comprising 50 patients with nr-axSpA, 40 with AS (20), 40 with RA, and 45 healthy controls. The clinical characteristics of patients and healthy controls are shown in **Table S1**. The results revealed that levels of anti-Kaiso antibodies were significantly higher in the nr-axSpA group than in the other groups (**Figure 2A**). We then sought to explore the differential expression of Kaiso proteins in tissues. Synovial and ligament tissues were collected from patients with transcervical fracture (healthy control, HC), AS, RA, and nr-axSpA patients who had ever undergone puncture biopsy of the sacroiliac joint and then sent for IHC analysis. IHC analysis revealed higher expression of Kaiso in synovial and ligament tissues from nr-axSpA patients compared with HC, AS, and RA (**Figure 2B**). Integrin subunit alpha 10 (ITGA10), as a key downstream molecule of Kaiso regulating osteogenic differentiation (21), was found to make no significant difference in the synovial and ligament tissues of patients with AS, RA, nr-axSpA, and HC (**Figure S3**).

### 3.3 Validation of the diagnostic value of the serum anti-Kaiso autoantibody level in axSpA and nr-axSpA

**Figure 3:**
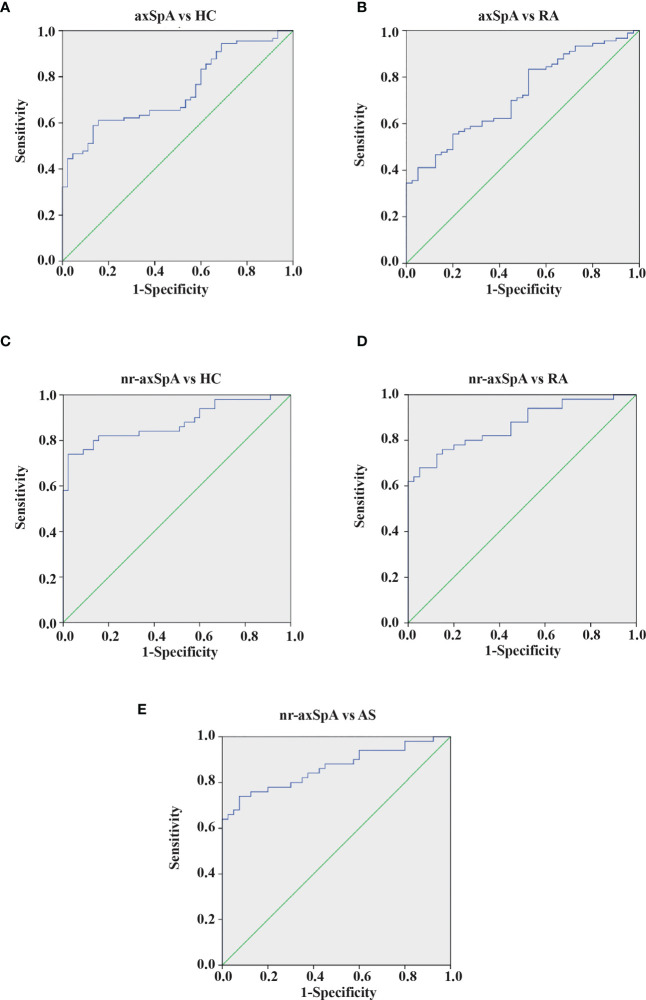
ROC curves of anti-Kaiso autoantibody for the discrimination of patients with axSpA from controls. **(A)** Patients with AxSpA (nr-axSpA and AS) versus healthy controls. **(B)** Patients with AxSpA versus RA. **(C)** Patients with nr-axSpA versus healthy controls. **(D)** Patients with nr-axSpA versus those with RA. **(E)** Patients with nr-axSpA versus those with AS.

The results presented suggest a possible role for anti-Kaiso autoantibodies as diagnostic biomarkers to discriminate nr-axSpA from AS and controls. We further analyzed the ROC curve of serum anti-Kaiso autoantibody levels to assess their diagnostic value (**Figure 3**). We found that serum anti-Kaiso autoantibody levels could differentiate patients with axSpA (nr-axSpA and AS) from healthy controls with an AUC (area under the curve) of 0.74 (cut-off value 4,065.49 ng/ml, sensitivity 61.1%, specificity 84.4%), and differentiate patients with axSpA from those with RA with an AUC of 0.72 (cut-off value 4,426.73 ng/ml, sensitivity 55.6%, specificity 80%). Using 4,574.98 ng/ml as the cut-off value, the AUC of anti-Kaiso autoantibody to discriminate nr-axSpA from AS was 0.86 (95% CI 0.78–0.94, sensitivity 76%, specificity 87.5%). Moreover, the diagnostic performance of serum anti-Kaiso autoantibodies in differentiating nr-axSpA from other groups (RA and healthy controls) was also assessed. The AUC value for differentiating nr-axSpA from healthy controls was 0.88 (with a cut-off value of 4,263.57 ng/ml, and a sensitivity of 80%, and a specificity, of 87%). The AUC value for differentiating nr-axSpA and RA was 0.87 (cut-off value: 4,574.98 ng/ml, sensitivity 74%; specificity 87.5%). The results of the ROC analysis are shown in **Table S2**.

### 3.4 Association between anti-Kaiso autoantibody and clinical characteristics

**Figure 4:**
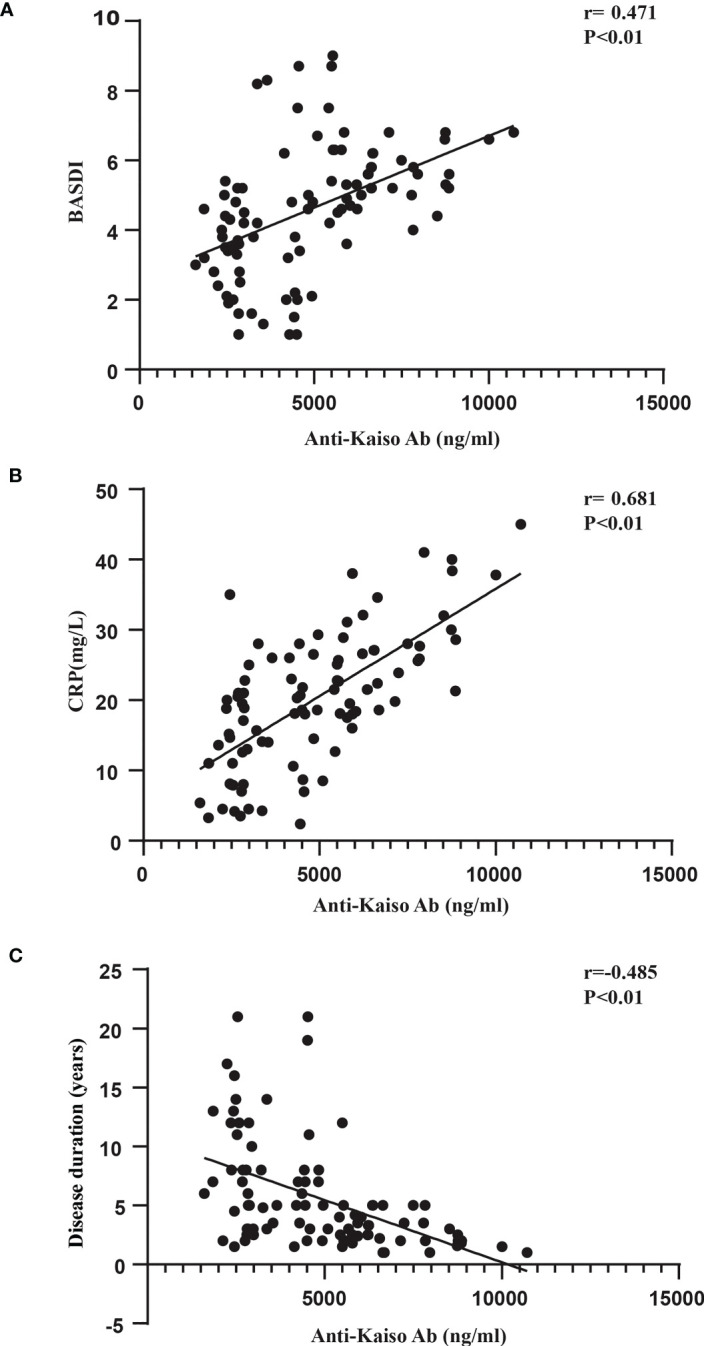
Correlation between anti-Kaiso autoantibody levels, BASDAI scores **(A)**, CRP **(B)**, and disease duration **(C)** in patients with axSpA.

Next, we sought to analyze the associations between anti-Kaiso autoantibodies and clinical indicators (**Figure 4**, **Supplementary Figures 1**, **2**). Of all patients with axSpA (nr-axSpA and AS), the serum level of anti-Kaiso autoantibody was positively correlated with CRP (r = 0.681, p <0.01) and BASDAI (r = 0.47, p <0.01) (**Figure 4**). However, it was not significantly correlated with other activity measures such as ESR (r = 0.131, p >0.05) and BASFI (r = −0.178, p >0.05) (**Supplementary Figure 1**). When further analysis was performed, the results showed that the anti-Kaiso autoantibody level was negatively correlated with disease duration (r = −0.485, p <0.01). Among nr-axSpA patients, the serum level of anti-Kaiso autoantibody was positively correlated with ALP (r = 0.456, p <0.01) (**Supplementary Figure 2**). Moreover, logistic regression analysis showed that anti-Kaiso antibodies (p <0.05), disease duration (p <0.05), CRP (p <0.05), and the BASDAI score (p <0.05) could discriminate “nr-axSpA” from “AS.”

### 3.5 Follow-up results of patients with nr-axSpA

**Figure 5:**
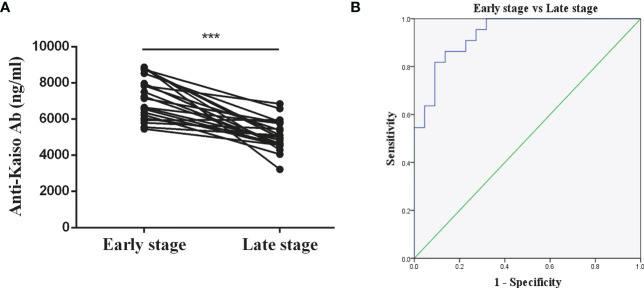
Diagnostic value of anti-Kaiso autoantibody in patients with AS. **(A)** Levels of anti-Kaiso autoantibody in patients with nr-axSpA progressing to AS over an 8-year period. **(B)** ROC curves of anti-Kaiso autoantibody for the discrimination of patients with nr-axSpA from those with AS. ***P <0.001.

Twenty-two of all the 50 patients with nr-axSpA progressed to AS over an 8-year period. According to **Figure 5A**, it was found that the anti-Kaiso autoantibodies of patients with nr-axSpA (7,233 ± 252.4 ng/ml) who progressed to AS (5,069 ± 178 ng/ml) were reduced to varying degrees overall. The AUC value for differentiating early and late stages of AS was 0.9 (cut-off value 5891.89 ng/ml, sensitivity 86.4%, specificity 86.4%) (**Figure 5**).

## 4 Discussion

The identification of biomarkers for early axSpA diagnosis and therapy is the current focus of research. By screening the serum of patients with nr-axSpA and controls using human proteome microarrays, we identified seven candidate autoantibodies that were present specifically in the serum of those with nr-axSpA. Considering the potential role of Kaiso protein in the inflammation and new bone formation processes of AS, an anti-Kaiso autoantibody was selected for further validation studies. The results demonstrated that serum levels of anti-Kaiso autoantibodies were higher in patients with nr-axSpA than in controls. We also found that anti-Kaiso autoantibodies were highly accurate in diagnosing nr-axSpA. Moreover, serum levels of anti-Kaiso autoantibodies were positively correlated with CRP, BASDAI, and ALP levels and negatively correlated with disease duration.

Autoantibodies are immunological hallmarks of several autoimmune diseases and can be used as diagnostic biomarkers (22, 23). AxSpA was long assumed to be a seronegative disease, and antibodies were not considered a hallmark of this disease. Recently, an increasing number of autoantibodies have been recognized as potential diagnostic biomarkers for AS (5). In this study, we demonstrated that Kaiso-specific autoantibodies are present in the serum of healthy individuals but at higher levels in the nr-axSpA. Interestingly, autoantibody levels returned to normal in AS. This phenomenon implies that the serum anti-Kaiso level may be correlated with the bone deformation stage in AS. We also found a positive correlation between the concentration of serum anti-Kaiso autoantibody and ALP among nr-axSpA in our study, which is supported by previous reports showing its relationship with structural damage (24). Moreover, Kaiso, an antigen of the anti-Kaiso autoantibody in tissues, may participate in the regulation of osteoblast differentiation. Therefore, both *in vivo* and *in vitro* experiments were designed to investigate the role of Kaiso in osteogenesis. The experiments indicated that Kaiso could inhibit osteoblast differentiation *via the* Itga10/PI3K/AKT signaling pathway. In addition, we discovered that the expression of Kaiso decreased the osteogenic differentiation of MC3T3−E1 cells and BMSCs through downregulating Itga10 (21). The higher expression of Kaiso in nr-axSpA patients also indicates its potential function. The likely reason is that, in the later stage of this disease, the previously normal target joints and surrounding tissues were gradually replaced by ossified tissue, which was associated with reduced expression of Kaiso. We also discovered that some other diseases are characterized by the disappearance of autoantigens during disease progression. For instance, Guillain–Barré syndrome (25), Hashimoto’s thyroiditis (26), and type 1 diabetes (27) are all autoimmune-related. As the disease progresses, its pathology is characterized by atrophy and necrosis of the target organs and tissues attacked by the immune system, which is accompanied by a reduction of some special autoantigens existing in them. These results may help to explain why anti-Kaiso autoantibodies were reduced in the AS stage. The following analysis revealed that serum anti-Kaiso autoantibody could be a potential diagnostic biomarker for differentiating nr-axSpA from other groups (AS, RA, and healthy controls). We further explored the diagnostic value of serum anti-Kaiso autoantibodies in the early stages of AS. We selected 22 patients with nr-axSpa who progressed to AS over 8 years and analyzed their change and clinical diagnosis value of anti-Kaiso autoantibodies. The results showed that the serum anti-Kaiso autoantibody level significantly decreased among patients with nr-axSpa with radiographic progression, which is suggested to be related to the progression of AS disease. However, most patients with AS have used biological agents, while those with nr-axSpA have not. Owing to different drug use in different groups, biological agents may have an impact on the immune process. Therefore, it is not known whether biological agents affect antibody production. However, this requires further experimental validation.

Several autoantibodies have been reported to be involved in osteoblast mineralization and bone formation in AS (9, 28). Kaiso (also known as zinc finger and BTB domain containing 33), a unique member of the poxvirus and zinc finger (POZ) family, exhibits dual-specificity DNA binding to methylated CpG dinucleotides or a non-methylated sequence known as the Kaiso binding site (KBS) (29, 30). Kaiso regulates inflammation and cell proliferation in various diseases (31–33). In addition, several studies have revealed that Kaiso affects Wnt signaling, which is essential for the regulation of bone homeostasis (34). Moreover, the zinc finger and BTB domain containing gene 16 (ZBTB16), which is also a member of the POZ-zinc finger family, regulates osteoblast differentiation (35, 36). Thus, we hypothesized that Kaiso plays an important role in axSpA structural damage. Bone destruction occurs prior to new bone formation in the pathological process of axSpA (8); therefore, it is possible that bone destruction in the focal axial joint during the early stage of axSpA is associated with increased Kaiso protein expression in the skeletal and connective tissues, and that the resulting increase in exposure to the immune system leads to increased levels of anti-Kaiso autoantibodies. The relative reduction in Kaiso expression in AS may contribute to the limited bone destruction and progressive new bone formation observed in these patients. Furthermore, immunohistochemical analysis revealed that the expression of Kaiso in synovial and ligament tissues of nr-axSpA patients were higher than that of AS, RA, and controls. However, no different expression of Itga10 was found between them. The possible reason was that anti-Kaiso autoantibodies interacted with Kaiso and interfered with its regulation of Itga10 expression. Thus, anti-Kaiso autoantibodies may represent a biomarker of nr-axSpA, although these findings have yet to be validated in more patients.

In addition to the anti-Kaiso autoantibody tested in this study, additional novel autoantibodies, including MYLK, EIF2C1, and SUGT1, are also potential biomarkers for nr-axSpA. MYLK gene activity can be induced by TNF-α, an essential inflammatory factor in AS (37). Additionally, EIF2C1 participates in angiogenesis (38, 39), whereas SUGT1 regulates inflammatory activity (40). Thus, all processes regulated by these genes appear to engage in distinct aspects of axSpA pathogenesis. Additional experiments are required to verify the role of these autoantibodies in axSpA.

In summary, the anti-Kaiso autoantibody can be a biomarker for nr-axSpA (especially in the early AS stage) and a potential therapeutic target. Further studies with larger sample sizes are required to validate our findings.

## Data availability statement

The original contributions presented in the study are included in the article/**Supplementary Material**. Further inquiries can be directed to the corresponding author.

## Ethics statement

The studies involving human participants were reviewed and approved by Ethics Committee of Shanghai Changhai Hospital. The patients/participants provided their written informed consent to participate in this study.

## Author contributions

YX and SZ collected the clinical data. XF and WT performed the experimental work and statistical analysis. XF and JL drafted the manuscript. WX designed the study and revised the manuscript. All authors contributed to the article and approved the submitted version.

## Supplementary material

Supplementary Figure 1 Correlation between anti-Kaiso autoantibody levels, BASFI scores **(A)**, ESR **(B)** in patients with axSpA.

Supplementary Figure 2 Correlation between anti-Kaiso autoantibody levels and serum ALP activity in patients with nr-axSpA.

Supplementary Figure 3 IHC staining of Itga10 in tissues of HC, nr-axSpA, AS, and RA.
