# Supplementary figures and images for: Diagnostic value of anti-Kaiso autoantibody in axial spondyloarthritis
Source: Front Immunol. 2023 Mar 30;14:1156350. doi: 10.3389/fimmu.2023.1156350 (PMC10098150; doi:10.3389/fimmu.2023.1156350)

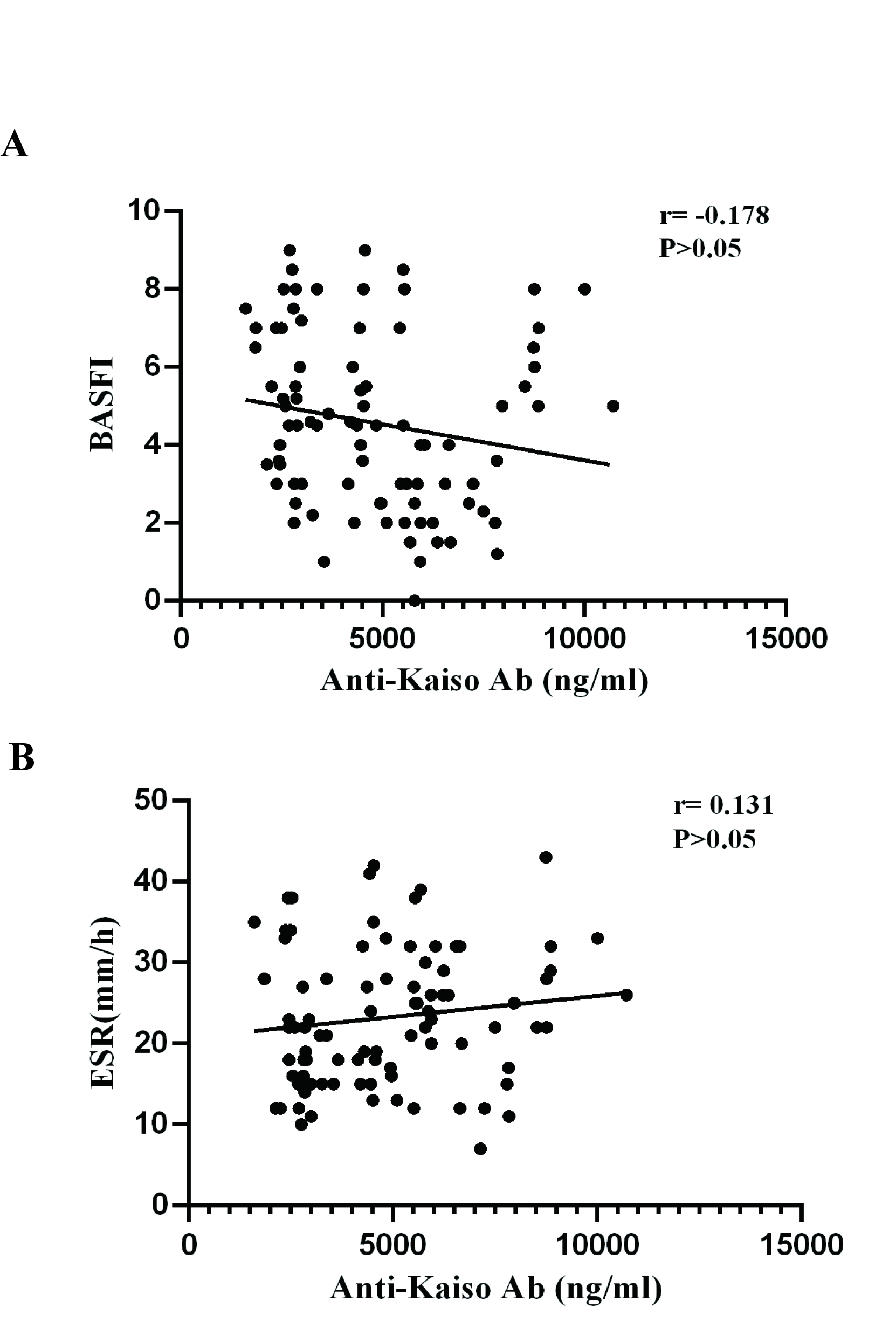

Supplement: Supplementary Figure 1 — Correlation between anti-Kaiso autoantibody levels, BASFI scores (A), ESR (B) in patients with axSpA. [file Image_1.tif]

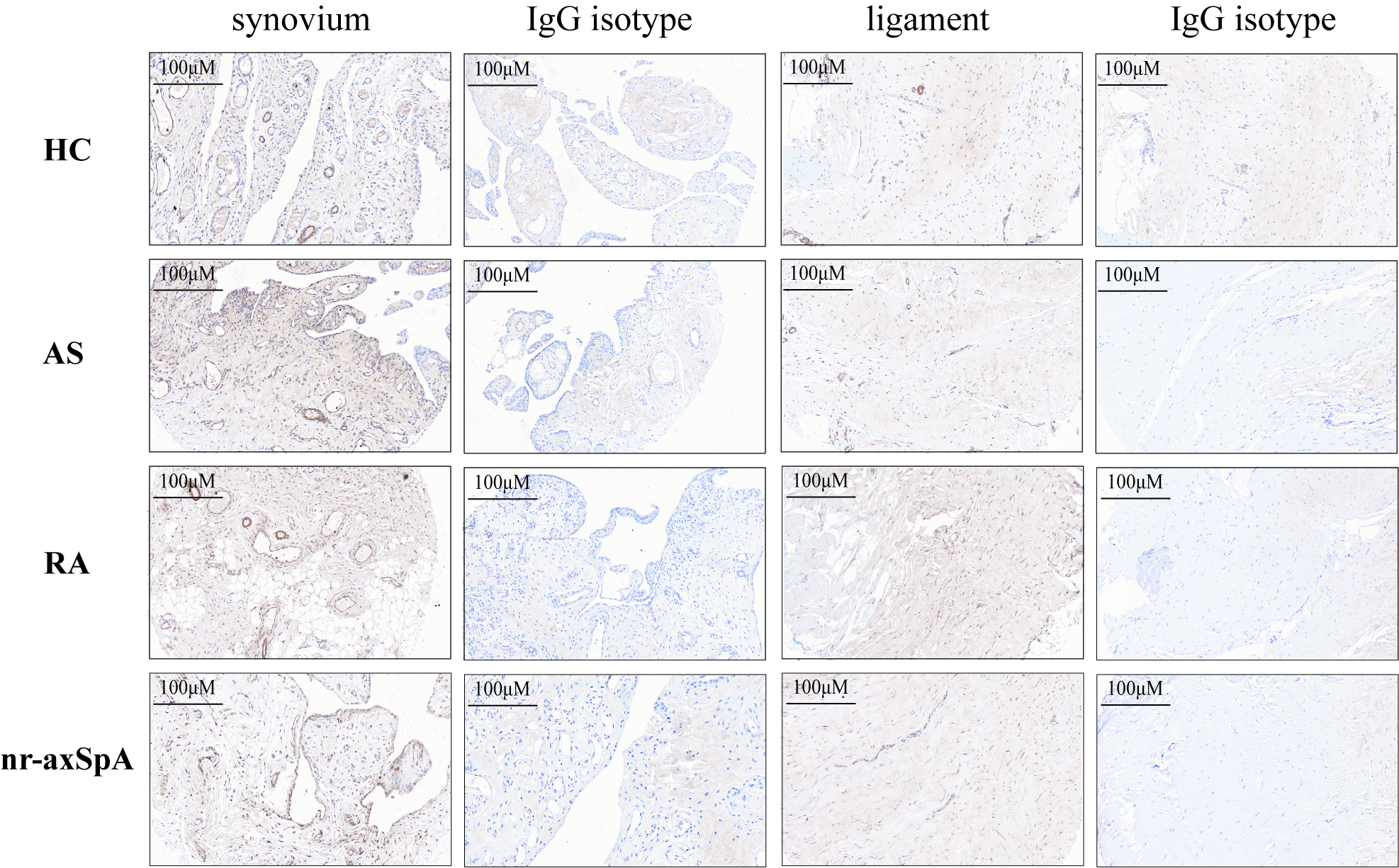

Supplement: Supplementary Figure 3 — IHC staining of Itga10 in tissues of HC, nr-axSpA, AS, and RA. [file Image_3.tif]
