# Supplementary material for: Diagnostic value of anti-Kaiso autoantibody in axial spondyloarthritis
Source: Front Immunol. 2023 Mar 30;14:1156350. doi: 10.3389/fimmu.2023.1156350 (PMC10098150; doi:10.3389/fimmu.2023.1156350)
Supplement: Supplementary file 4 [file Table_1.docx]

Table S1. Clinical parameters of patients for Meso Scale Discovery assay

|  | HC  (n=45) | nr-axSpA  (n=50) | AS  (n=40) | RA  (n=40) |
| --- | --- | --- | --- | --- |
| Age, years | 33.67±9 | 32.28±8 | 39.8±9.76 | 40±6.56 |
| Gender, male, n (%) | 25 (56) | 37 (74) | 33 (83) | 15 (38) |
| Disease duration, years | NA | 2.75±1.18 | 9.28±4.86 | 5.76±2.79 |
| ESR, mm/h | NA | 21.54±7.79 | 25.35±8.80 | 23.63±8.9 |
| CRP, mg/L | NA | 24.07±8.56 | 14.95±8.44 | 13.79±5.36 |
| HLA-B27 positive, n (%) | NA | 43 (86) | 34 (85) | NA |
| BASDAI score | NA | 4.87±1.55 | 4.19±2.18 | NA |
| BASFI score  mSASSS score | NA  NA | 3.30±1.77  NA | 6.15±1.63  17.42±10.09 | NA  NA |
| Treatment, n: | NA |  |  |  |
| NSAID |  | 38 | 10 | 13 |
| DMARDs |  | 9 | 15 | 17 |
| Anti- TNF-α |  | 0 | 15 | 10 |
| Sacroiliitis grading, n: | NA | NA |  | NA |
| GradeⅡ |  |  | 11 |  |
| GradeⅢ |  |  | 18 |  |
| GradeⅣ |  |  | 11 |  |
| Spinal involvement | NA | NA | 40 | NA |

Abbreviations: ESR, erythrocyte sedimentation rate; CRP, C-reactive protein; NSAID, non-steroidal anti-inflammatory drug; DMARDs, disease modifying antirheumatic drugs; NA, not applicable; Data are expressed as the mean±SD.
