# Supplementary material for: Diagnostic value of anti-Kaiso autoantibody in axial spondyloarthritis
Source: Front Immunol. 2023 Mar 30;14:1156350. doi: 10.3389/fimmu.2023.1156350 (PMC10098150; doi:10.3389/fimmu.2023.1156350)
Supplement: Supplementary file 5 [file Table_2.docx]

| Diagnostic groups | AUC  (95% CI) | p value | Cutoff | Sensitivity  (%) | Specificity  (%) | PPV | NPV |
| --- | --- | --- | --- | --- | --- | --- | --- |
| A+B vs C  A+B vs D | 0.74  (0.66-0.82)  0.72  (0.63-0.81) | <0.01  <0.01 | 4065.49  4426.73 | 61.1  55.6 | 84.4  80 | 88.7  87.9 | 47.9  55.6 |
| A vs C | 0.88  (0.80-0.95) | <0.01 | 4263.57 | 80 | 87 | 87.0 | 80.0 |
| A vs D  A vs B | 0.87  (0.80-0.94)  0.86  (0.79-0.94) | <0.01  <0.01 | 4783.73  4574.98 | 74  76 | 87.5  87.5 | 88.1  88.4 | 72.9  74.5 |

Table S2. Diagnostic performance of anti-Kaiso autoantibody

A, nr-axSpA; B, AS; C, Healthy control; D, rheumatoid arthritis; CI, confidence interval; PPV, positive predictive value; NPV, negative predictive value.
